# "The Hospital" Nursing Mirror

**Published:** 1899-06-10

**Authors:** 


### "The Hospital" Nursing Mirror.

Being the Nursing Section of "The Hospital."

[Contributions for this Section of "The Hospital" should be addressed to the Editor, The Hospital, 28 & 29, Southampton Street, Strand, London, W.C., and should have the word "Nursing" plainly written in left-hand top corner of the envelope.]

### Notes on News from the Nursing World.

### THE ROYAL RED CROSS.

The principal item of interest to nurses in the Birthday *Gazette* on Saturday was the announcement that the Queen has been graciously pleased to confer the decoration of the Royal Red Cross upon Miss Isabella Smith, of the Royal Naval Nursing Service. This great honour has been bestowed upon Miss Smith in recognition of her devotion and competency in connection with her nursing duties in naval hospitals at home and abroad and during the Benin Expedition. On the Queen's birthday Sister M. E. Harper, of the Army Nursing Service, who worked hard in Egypt, and who has since been stationed at Malta, was decorated by the Governor, Sir Francis Grenfell, with the Royal Red Cross, immediately after the Royal salute had been fired at the Battery.

### THE ROYAL BRITISH NURSES' ASSOCIATION.

The Princess Christian will preside at the annual meeting of the Royal British Nurses' Association on Saturday afternoon. We remind the members of the corporation that the meeting will take place in the Westminster Town Hall at three p.m., and that the business on the agenda is: To receive the annual, financial, and other reports; to elect vice-presidents; and to elect the general council. After the meeting there will be a pleasant reunion of members and their friends in the grounds of the Earl's Court Exhibition. Full particulars and tickets may be obtained from the Secretary of the Association, 17, Old Cavendish Street, Oxford Street, W.

### THE NEW HOSPITAL AT WOODFORD.

It is to private munificence that the inhabitants of Woodford and the surrounding district are indebted for the handsome new hospital which was opened last week by the Duke and Duchess of Connaught. Mr. J. R. Roberts has expended no less than £8,000 on the undertaking, and Mr. W. H. Brown, by a contribution of £600, completed the endowment fund. In declaring the institution open, the Duke of Connaught expressed his gratification that, being erected in commemoration of the Queen's Diamond Jubilee, the hospital was so closely identified with the name of his beloved mother. A picturesque feature of the function was the presentation by Miss Ivy Isitt, niece of the donor of the hospital, of a beautiful bouquet to the Duchess. The tiny mite was attired in nurse's costume, and gravely curtsied to the visitors as she made her way on to and off the daïs.

### THE KENT NURSING INSTITUTION.

The difficulties which threatened to wreck the prospects of this institution having, happily, been finally overcome, Miss Ligertwood and the 23 nurses who have stood by her during the progress of a dispute (into which we need not enter) have settled down to their useful and admirable work at West Malling. Thanks are certainly due to Mr. Herbert Leney, whose influence as an intermediary was exerted with the most excellent results. It is, of course, well known in the district that the staff under Miss Ligertwood had intimated that in the event of her resignation it would be impossible for them to remain. Moreover, they had decided to ask her to open an institution on co-operation principles, and in order that she might have no financial risk had subscribed £500, and placed it at her disposal so long as she required it, promising also to serve her loyally and obediently. A superintendent who is capable of arousing such devotion as this cannot fail to possess both the capacity and the judgment which are indispensable in such a position, and we warmly congratulate the subscribers to the Kent Nursing Institution and the public on the fact that Miss Ligertwood is to remain at the head of the organisation to whose success she has so materially contributed.

### THE CHILDHOOD SOCIETY.

Lord Egerton of Tatton presided at a meeting of the Childhood Society at the Sanitary Institute Conference on Friday. A discussion upon "the education of children feebly gifted under the care of guardians" was opened by Dr. Shuttleworth, who was followed by Mrs. Burgwin (Superintendent of Special Instruction, London School Board), Miss Lidgett and Mrs. Warner (Poor Law Guardians), Dr. Rhodes, Mr. Hoskins (Kensington and Chelsea Banstead Cottage Homes), and Mrs. Maitland. All the speakers were agreed upon the extreme desirability of giving mentally deficient or "feebly gifted" children specialised instruction under trained teachers. Dr. Shuttleworth urged that such children should be removed as much as possible from poor law surroundings, and that the school age should be more or less indefinitely prolonged instead of terminating at sixteen. Mrs. Burgwin insisted upon the importance of studying the causes which lead to a feeble-minded condition, and advocated prevention rather than cure, and that by means of extended control these children should never become parents themselves. Miss Lidgett said she believed that the want of sleep and want of proper food common amongst children of the very poor produced feebleness of intellect. Both Miss Lidgett and Mrs. Warner quoted from their own experience cases showing the terrible harm consequent on the unfettered liberty of action allowed to these unfortunates. Other speakers gave encouraging accounts of the success of special classes where they had been adopted, a very general feeling prevailing that any extra expense incurred was amply compensated for by the result. It was recommended that unions should combine for the purpose of providing specialised treatment of the "feebly gifted" under their care.

### THE ANGLESEY NURSING ASSOCIATION.

It is satisfactory to learn that the money collected towards the support of the Anglesey Queen's Jubilee Nursing Association, initiated eighteen months ago, exceeds £1,000. This, as the chairman observed at the annual meeting, is a good nucleus; but he also expressed his confidence that, as soon as the work of the nurses is appreciated, more help will be forthcoming. Nurses have recently been appointed, under the auspices of the association, in several districts. It is a pleasing feature that several of the churches and chapels subscribe £5 for five years. The grants made by the association are, of course, supplementary to local resources.

### THE NEW STATION AT DROITWICH.

THOSE nurses to whose lot it may fall under doctors' orders to take patients to Droitwich in the hopes of benefiting them by the salt baths, will rejoice to hear that the new station, which was opened amid general rejoicings on Saturday, is a distinct improvement on the old uncovered structure that formerly extended anything but a welcome to fresh arrivals. In fact, the draughts which played around were suggestive rather of fresh chills to nervous rheumatic invalids. How much the town has developed of late is shown by the statement that, whereas 35 years ago seven trains a day sufficed for the accommodation needed, over 60 are now required; and probably the fresh conveniences offered will result in a large increase of visitors. The brine baths have a remarkable effect upon many diseases, though there is little going on in the way of excitement. Apparently, lest visitors should look back to gayer scenes with regret, St. Peter's Church has an inscription written over the entrance, "Remember Lot's Wife."

### TRAVELLERS AND PHTHISIS.

THE question of the isolation of consumptive patients on ocean steamers continues to excite great interest. A correspondent, writing from personal experience, says: "Those who do not travel will scarcely realise what it means to be put into a small cabin containing four berths (so small that the occupants can with difficulty dress two at a time), and find that one of your fellow-passengers is in an advanced stage of consumption, and is going, say, to Egypt for the winter, having been told that it is his only chance to prolong his life till next summer. This poor patient—for no one can call him by no other name—is on deck during the day, but, being weak, retires early to rest and does not leave his bed till late in the morning. During that time he is coughing and expectorating continually, to the distress of the other occupants of the cabin, who, being perhaps afflicted with *mal de mer*, are confined to their berths more closely than they could wish. Added to this, to say the least of it, unpleasant state of things, very little fresh air enters the cabin, and the atmosphere below decks is frequently stifling. If the consumptive person's destination is Egypt this kind of thing will go on for a fortnight, and then he will land. In all probability his berth is already bespoken for a passage to India, and with only a change of sheets, the new occupant takes the consumptive's place, which has been vacant but a few hours. It is needless to mention that clothes of any kind are rarely washed on board, and the soiled linen of consumptives, as well as of others, is kept in the cabin till the end of the voyage, unless it is old and thrown overboard as worthless. The number of germs that infest a ship's berth is too horrible to contemplate."

### DEATH OF RUSSIA'S FIRST LADY DOCTOR.

MADAME BARBARA ALEXANDROWNA KASCHENMAROWA-NUDNEWA, the first lady permitted to qualify for the medical profession in Russia, died last week after labouring for thirty years in the Ural district amongst the Cossack women. Having represented to the Minister of War the necessity for a female doctor for the Cossack women, Madame Kaschenmarowa-Nudnewa bravely faced, and finally overcame, the obstacles placed in her way. At the age of thirty she had passed all her examinations with honour. On qualifying, she immediately retired to the Ural district, and commenced to practise as a doctor. The event caused great sensation in Russia. By her skill, devotion, and diligence she not only made a name for herself among the Cossack women, by whom she was greatly trusted and loved, but opened the way for her sisters, many of whom have since qualified as doctors. There can be no subject of overcrowding the medical profession in the Muscovite empire at present. Some districts of 100,000 inhabitants are rich in possessing one doctor and one midwife, that is to say, rich in comparison with other vast districts which have neither the one nor the other.

### A QUESTION OF IMPORTANCE TO PROBATIONERS.

THE Hospital Committee of the Newcastle Board of Guardians decided the other day against giving the probationary nurses in the workhouse training in midwifery during the third year of training in the "house." It is alleged that the decision amounts to a breach of faith with the probationary nurses, and the matter has been referred back for further consideration to the committee. The plea of the latter is that there are not a sufficient number of births in the workhouse to give adequate training. There may be force in this contention, but at any rate there can be no excuse for further misunderstanding. A probationer entering the "house" for training has a right to know exactly what she will be trained in, and it is due to her that she shall enjoy the advantages of the precise kind of experience which was promised her.

### EXPERIENCES OF A LADY DOCTOR IN LUCKNOW.

A MEDICAL woman who has been visiting some of the hospitals in India, mentions in the *Zenana* a curious episode. "One night visit," she says, "I paid with Dr. Ferguson to a native Christian patient in a slum of Lucknow I shall never forget, for it had its amusing as well as its serious features. The patient's husband entirely misled us as to the nature of the case, which turned out to require instruments we had left at the hospital. So we had to wait anxiously in a small hut, only big enough to hold two beds and a stool, while the mendacious husband did penance by going all the way back to fetch a necessary bag. The only light was a tiny lamp—some oil in a saucer, with a wick floating in it. . . . . . As we drove back the next day from a visit to the same patient, various people rushed from the little side streets of the bazaar, calling us to stop and see some sick persons—one old man, who knew some English, ran by the side of the mission carriage, thrusting a small child towards us and crying out, 'Mother dying, mother dying.'"

### LITTLESTONE-ON-SEA CONVALESCENT HOME.

ANY information respecting convalescent homes is welcome at this season of the year, and many will doubtless be glad to hear of the convalescent home at Littlestone-on-Sea in Kent. This home is open all the year round for the reception of women and children of humble station. One great advantage is that a letter of admission may be purchased for £1 1s., which entitles the holder to send an adult for a fortnight, or a child for three weeks, free. The Charity Organization Society has made use of this home for some years past. At present beds are provided for 31 patients, but preparations are being made to increase the accommodation in order to enable the authorities to receive 50. The patients are in charge of a matron, and are required to make their own beds and assist in the lighter domestic duties of the establishment.

### SKIPPING AS AN EXERCISE FOR NURSES.

A correspondent, writing from Cardiff, says: "I wonder if many nurses are aware of the value of skipping as an exercise. Some, I know, will laugh and ask what time or opportunity they can have for such nonsense, but if they realised how skipping on tip-toe strengthens the muscles of the calf and foot, which so often give way and cause that much-dreaded flat foot, they would, at least, consider it. When first I began nursing I suffered very much with my feet, as many nurses do, and after about three months in a small hospital I had to keep them constantly bandaged for the greater part of the rest of that year, otherwise I could not have kept about. Some years after, before entering a large London hospital, I consulted a doctor as to whether I could not strengthen my feet and ankles. He advised skipping, at first for five or ten minutes a day, gradually increasing the time to an hour, douching with cold water and no bandages, as of course the muscles learn to depend on them instead of on themselves. The benefit I gained was so great that even with the much harder work of a London hospital my feet 'went' without support of any kind. I believe many girls who are thinking of a hospital training would be saved suffering from flat foot if they would try to strengthen their feet for some weeks before beginning work, and even after. Even nurses might steal at least a few minutes in the day from tennis or cycling, which I know some find time and opportunity for, and use the skipping rope."

### NURSING AT NEWLYN.

Although there has been a nursing society at Newlyn for some years, its prosperity is not so great as it ought to be in a well-to-do village is famous for having originated a school of artists. In consequence of this state of the funds no nurse was employed between November and the spring mackerel season. But while it is true that Newlyn—where more than a million mackerel were landed last week—is exceptionally healthy, it is large enough to keep a nurse always going; and neither artists nor fishermen are likely to enjoy immunity from accident or indisposition. There was a collection for the nursing fund by ladies on the pier the other day, and we hope that the response of the fishermen and artists was satisfactory.

### A COUNTY HOSPITAL WITHOUT A TRAINED MATRON.

Incredible as it may seem, there is in one of the home counties, not 50 miles from London, a county hospital which does not possess a trained matron. It was purely by accident in the first instance that this state of things arose; but it has continued for ten years. The result, we gather from a correspondent, who names the hospital, is not satisfactory. There is a lack of sympathetic interest in the work of the nursing staff, a want of conformity to etiquette, and an absence of discipline, which would scarcely prevail under the usual conditions.

### THE SUPPLY OF NURSES IN IRELAND.

Some interesting particulars of a number of the principal provincial hospitals and infirmaries are given, along with numerous excellent illustrations, in the *Englishwoman* for this month. Mention is made of the nurses' home and training school in connection with the Belfast Royal Hospital, which provides nurses for the hospital and for private nursing. The applications, it is stated, average about 200 a year for admission as probationers, and the vacancies do not average more than 18 a year. By this we judge that there are plenty of Irish young ladies anxious to become nurses. The training at Belfast only lasts a year and a half, but candidates are required to bind themselves to remain another two years in the service of the home, either as private nurses or to serve in the hospital. A premium of £10 is exacted, and there is no salary given the first year. For the second year a salary of £14 is paid, and this gradually increases up to £26 for the nurses. The sisters receive £36. Separate bedrooms are provided for the staff nurses, but not for the probationers.

### THE CARE OF CHILDREN AT ADELAIDE.

Perhaps it may interest some nurses who are specially devoted to children to know what care is taken of the small sick folk at the Antipodes. The children's hospital at Adelaide is most charmingly situated in the highest part of the city, with beautiful grounds and lawn, where the invalids are carried out into the sunshine and air. The wards are cheerful and pretty, and quite up to date. There is an ingenious contrivance for flowers at the foot of each cot, invented by an enthusiastic lady visitor. It is a kind of wire basket hanging from a rail, with a vase or bowl fitted into the basket, which is always filled with garden or wild flowers, and is just in the position where the child can enjoy them. The effect of these baskets full of native orchids, maidenhair ferns, ericas, or buttercups and marguerites, and the various flowers and grasses which abound in that part of the world is most pleasing, and it is a real source of joy to the children who spend a long time in bed to have a little colour and variety brought into their lives.

### SHORT ITEMS.

On Thursday afternoon next week Mr. Victor Horsley will give an address at the West Ham Hospital, Stratford, upon the work of the General Medical Council "with special reference to the proposed registration of midwives."—At the yearly gathering last week in connection with St. Monica's Home Hospital for Sick Children it was stated that 71 patients had been under treatment in the institution during the year. There was a religious function, after which the guests of the committee were taken over the wards of the hospital.—The Duke and Duchess of York have arranged to open the new homes of the National Society for Employment of Epileptics at Chalfont St. Peter on Friday, June 23rd, instead of June 22nd, as previously announced.—"The Stock Exchange Ward" of the Royal Eye Hospital, Southwark, adding 14 new beds to the institution, was opened by the Chairman of the Stock Exchange on Tuesday, in the presence of a large company. Mr. Hichens, in a brief speech, said that all who were engaged in the City, where their eyes had to be very much used, and oft-times tried, had much to thank the ophthalmic surgeon for.

### District-Visiting Nursing in Obstetric Practice.

By A. Worcester, M.D., Waltham, Massachusetts.

(*Concluded from page* 127.)

Vaginal examination of patients in labour is absolutely essential in district-visiting nursing. But nurses are often employed in families where the doctor is expected to be dancing in attendance, and he, rather than the nurse, is depended upon for such examinations. In Waltham physicians have come to depend upon the nurse's knowing how in this way to keep watch of the labour and to report its progress, while physicians elsewhere can hardly believe that nurses can be depended upon for such work; and, indeed, some otherwise fine nurses teach to the contrary, in their ignorance maintaining that such work is not proper for nurses. Nevertheless, the custom will surely grow in spite of what physicians and nurses feel and say. It properly belongs to obstetric nursing. The necessity of physicians running into the house often during labour exists only where the nurse is incapable of informing him when he is needed. At the beginning of the century it was considered outrageous for any man physician to attend women in labour. That was left to the midwives. It was because they were so uneducated that the man obstetrician has come into fashion, but there is no intrinsic fitness in his serving as a watchful nurse at the bedside forty-eight hours before he is wanted.

In order to examine properly it is necessary first to have the patient out at the right side of the bed, well on to the edge, hips firmly placed, their weight on left trochanter resting firmly on the bed where supported by the sideboard of the bedstead. The left thigh should be flexed forty-five degrees. The right thigh will be steadily supported and properly flexed if the right foot is caught under the hollow of the left knee. With the thighs in this position the axis of the vagina is then in the direction of the left thigh. Thorough scrubbing with soap and water, and afterwards in corrosive sublimate, 1 to 1,000, while it does not prepare hands for handling ligatures or for wringing out sponges for abdominal operations, prepares well enough for vaginal examinations. There is no need of any lubricant if the vulva is properly separated by the fingers and thumb of the left hand before inserting the two first fingers of the right hand. Sterile vaseline might be used, but for repeated examinations even that is objectionable, because very hard to wash off, and it catches all the dirt it encounters. In separating the vulva for examination, pass the left hand in front between the thighs. Such examinations ought to be made in plain sight of what one is doing. Always enter the vagina posteriorly, keeping well to its posterior wall, with two fingers in line with the long diameter of the vulva from front to back, rotating arm and shoulder as you lean forward over patient, so that the ball of the finger comes forward against the surfaces to be examined by touch. Explore one quarter after another. Remember that what is wanted is mainly information as to the os, the condition of the cervix, and the presentation.

If the physician has not already visited the patient, the nurse, as soon as possible after her arrival at the case, should send him a note describing the condition of the patient and the progress already made. Unless otherwise instructed, it is her further duty in normal cases of labour to send for the physician when the first stage is nearing its end. The nurse is therefore responsible for the proper summoning of the physician, and in the meantime for the assurance to patient and physician that all the conditions are normal. Proper training and experience amply qualify the nurse for this service.

I pass over the nurse's duties during the second and third stages of labour, as the physician's assistant or, in case of his absence, as his lieutenant. In this paper I want to set forth the especial duties of the district nurse in midwifery service.

In washing up after labour no intra-vulva washing is permissible. The line between the mucous membrane and the skin limits the nurse's service. Any intra-vulva cleaning must be done by the physician or under his orders. I remember some years ago a nurse being called to account very severely by one of our famous obstetricians because on the labia minora, when perineal stitches were being removed later on, was found some of the vernix caseosa. That nurse would now be applauded. It would be officious and reprehensible for the nurse to attempt to scrub off that tenacious stuff. What better dressing could be applied? The line limiting the nurse's province may be found by holding the labia majora together while washing the vulva. In this way thorough cleansing can be effected without hurting or harming the patient.

If the bed has been properly made and the patient properly prepared it is astonishing how little needs to be done afterwards in removing every trace of the labour. After washing the patient and once rolling her half over, the sheet-skirt and the provisional draw-sheet can be removed, the binder and napkin applied, the nightdress brought down into place, and all this in a very few minutes. I need not picture the confusion, the fuss and bother to all concerned where the clothing of both patient and bed has all to be changed. Nor can I now consider the great advantages to the baby in being first washed and dressed by a nurse who among other things knows enough not to fill its eyes with soap-suds, nor to stick the belly-band pins through its skin and then to give it gin to stop its howling. But let us hurry on to the consideration of the blessings to both mother and child that the district nurse can bring on her after-visits.

Were it necessary for the poor woman to choose between the service of a district-visiting nurse for an hour or so each morning for the week of her confinement and the calls of the physician, there can be no question which she should choose. For almost all the skilled nursing she needs can be given her on such calls, and for her care during the rest of the time she can safely depend upon her sister or daughter or friendly neighbours. Indeed, it is in the instruction of these helpers that the district-visiting nurse has almost her greatest opportunity for good. They can be taught how to make the gruel and broth, how to dress and undress the baby, and how to do the thousand and one services needed in the lying-in room.

The especial service of the trained nurse in confinement cases lies in precaution-taking, in the prevention of troubles. But for this it is not necessary that she shall be on constant duty at the case. It is enough, for instance, in the proper care of the nipples and breasts if she teaches the patient how to bathe and anoint the nipples, and if at the proper time she bandages the heavy breasts. And so in the care of the bladder. If eight or ten hours after the labour the nurse visits the patient and helps her to empty her bladder, the danger of retention and catherisation is much diminished. The same is true in the care of the rectum. And indeed, over the whole range of the lying-in period, most of the usual suffering and trouble can be prevented by proper nursing, and most of this advantage can be had where only district-nursing visits can be afforded.

To the very poor woman who otherwise could not have any nursing, and at most only the occasional help of a kindly neighbour, the district nurse comes as an angel from heaven. During her labour she need not suffer the inquisition of the neighbourhood, and during the following days she can at least rest in bed.

But, after all, it is not only to the very poor mothers and babies that district-visiting nursing comes as a blessing. It is neither for the submerged tenth, who cannot have either the luxuries or the necessaries of life, nor for the sublimated tenth of the population who can have everything except contentment, that there is the greatest need for planning improvements. Our chief responsibility is rather to the great middle class of self-respecting families of moderate means who pay for everything they have and yet can afford but few luxuries. To such families the maternity service of district nursing is indeed a boon. Instead of the cheap, untrained nurse, who fails both as nurse and house servant simply because she tries to serve in that dual rôle, a perfectly trained nurse can be had for the labour and for the few hours on following days, that is, for only such time as trained nursing service is needed. By paying full price to the district nurse by the hour, and by paying servant's wages to a servant, the total cost is less than for the service of even the cheapest kind of nurse who undertakes to do everything.

Inasmuch as in district-visiting nursing there is such grand opportunity for acquiring experience, which, after all, is the professional nurse's real capital, nurses are very willing to work in it for far less money return than they rightly expect when half their time is spent in waiting, and much of the remainder in unnurse-like work or idleness in wealthy homes.

Wherever introduced this sort of nursing will surely take deep root, because

(1) Nurses themselves recognise the opportunities so afforded for advancing in their profession.

(2) Physicians once accustomed to the assistance of such nursing in their midwifery practice will never be long without it.

(3) Families soon learn so to depend upon visiting-nursing service that the demand for it becomes steady and strong.

### St. Mary's Hospital Bazaar.

THE bazaar in aid of the fund for completing the Clarence Wing of St. Mary's Hospital took place on Tuesday and Wednesday. It was held at the Hotel Great Central, the proprietors having kindly given up the whole of the ground floor to the function, a most praiseworthy way of celebrating the opening of the hotel itself. The stalls were picturesquely arranged in the handsome entrance-hall, and were named after well-known streets and squares. Amongst the stall-holders were the Princess Adolphus of Teck, the Countess of Ilchester, Lady Roberts, Lady Broadbent, Lady Dimsdale, Lady Jeune, and Lady Sassoon, who owing to the heat did a roaring trade in American drinks. The Duke of Fife on Tuesday alluded pathetically to the pleasure it gave his wife to open the bazaar, because her own poor health so often prevented her from taking part in public ceremonies.

A nurse at St. Mary's sends us what she happily calls "a few side lights" on the work of preparation which has been going on in the hospital for some months prior to the bazaar: "When first the bazaar was spoken of we all felt a vague outside curiosity as to how it would succeed, but it never occurred to us that we, too, should be called upon to help to build the 'New Wing.' Of the want of beds we were only too conscious. Many of us had watched the faces of the people, when having brought their sick ones in certain hope of admission, the sorrowful words had fallen on their ears, 'No room,' and the sight of their overwhelming despair had often made us long for help for these sufferers.

"The first faint dawn of hope came ten years ago, when the 'Clarence Memorial Wing' was put before us, and plans for helping came clear and straight. One and another grasped the situation and stepped to the front, so that the ground floor was completed, but the ground floor only. Then one Saturday afternoon, just nine weeks ago, I was presented by the home sister with a small yellow collecting card. I took it, looked at it, turned it over. Collecting cards I have always utterly abhorred. One grand thing, it was small and had only ten spaces. I could see I was expected to fill it. Well, that very afternoon, while I was waiting to go to the surgical out-patients, I passed my card round. I must tell you about the first giver, a little pupil-teacher of fourteen years. She gave me a shilling, and when she went home she begged all her friends for pennies for the new wing, and the next week brought me three and sixpence. Then she went down to the country, and while she was away made two pretty pinafores for the bazaar.

"All the out-patients seemed to grasp our great need at once. I never mentioned the bazaar, but went in altogether for the extra beds. The women specially responded. I did not care to ask the men. The medical patients seemed to wear such a down look, but the surgical and the morning casualties were my best contributors. A man who had given me many pennies called me aside one morning and he told me he had an only daughter, a girl clever at her needle, and a dressmaker by trade. She felt, he said, that she would like to work something for the bazaar just as a thankoffering for her father's recovery. Last Saturday he came armed with twelve dainty pincushions; his daughter had risen early and sat up late to get them finished in time. We took him to the matron, and he was so pleased and so proud. Such sympathy and willingness to help from the patients cheered us up and encouraged us to go on.

"One day I was not able to collect, and some small children sent me five half-pence. I found out that each brick cost threepence, so the children used to go shares in buying one; I had often four farthings in a share.

"Some of the convalescent patients, aided by the Sister, have toiled hard for months, and piles of things have come out of some wards. Dolls dressed as patients with little labels tied on, 'Please, I want to come to the Clarence wing.' One on a tiny trolley, ready to start for the operating theatre, fully equipped, a carbolic dressing on the leg, and a tray of antiseptic dressings close at hand. A sister was in readiness to accompany this patient. Another doll, dressed as a matron (by an old lady over eighty) looked very fine. A blind woman knitted some babies' socks; one of the nurses made a beautiful frock for a baby in her off-duty time; and another carved a little table. The Duchess of York sent a newspaper rack, done in poker work, which was quite restful to look at—cows, water, trees, a ship, and a sunset sky."

### A Nurse's Day of Rest.

THE annual "Day of Rest" for the nurses of the metropolitan hospitals and nursing institutions was renewed at Hertingfordbury on Thursday last. A considerable number of nurses were present. The "Day" commenced with a celebration of the Holy Communion in the parish church at eleven. The rector, Canon Burnside, was celebrant, assisted by the Rev. A. G. Locke, chaplain of St. George's Hospital; and the Rev. T. H. Russell, chaplain of the North-Western Hospital. The Bishop of Southwark gave an address upon the responsibility of using such seasons of rest and retirement for the recreation of body, mind, and soul. A second service followed at half-past three, when the Bishop dwelt upon the operations of the Holy Ghost with special reference to the nurses' vocation as a ministry of healing. The nurses were entertained at dinner and tea. The intervals between the services were mainly spent in rambles in the country. It was generally felt that the "Day" had not failed in its principal intention of affording the nurses the opportunity of physical rest combined with spiritual edification.

### The Nursing of Phthisis in Sanatoria under the "Open-Air" System.

By the MATRON of the National Sanatorium for Consumption, Bournemouth.

### III.—THE QUESTION OF EXPECTORATION.

RECOGNISING, as we do, that consumption is a preventable disease contracted by taking the germs of tuberculosis from the dust of dried sputum into the system, great attention must always be paid to the collection and destruction of all expectoration. The proper education and training of the patients in this direction of necessity falls very largely into the nurses' hands, and it is of such paramount importance that too much stress cannot be laid upon it. After several experiments, it has been decided by the physicians attached to this sanatorium to strictly forbid the use by patients of handkerchiefs of the usual style, those of the Japanese or paper variety being substituted. The latter are prepared by the majority of purveyors of nursing requisites and cost about 1s. 6d. per 100, some houses making a reduction on quantities of 1,000. In cases when there is much nasal catarrh these are of little use, as they become soft and pulpy if thoroughly wetted, and the nurse will find squares of old linen answer the purpose better; in either event she should be careful herself to collect the temporary handkerchiefs each morning, or oftener if necessary, and see them burnt in the furnace, otherwise patients are very liable to leave them about or throw them away out of doors.

In a modern sanatorium patients are forbidden to spit about in the grounds and all public places under pain of immediate dismissal, and every means are taken to inculcate in them habits of cleanliness and so abolish a dangerous and filthy habit. They are carefully instructed on no account to swallow their own saliva. In order that these regulations may be carried out satisfactorily each patient is provided with a Dettweiler's Pocket Spittoon Flask. This spittoon is made on the principle of an unspillable inkstand, of thick blue glass, with removable metal inside funnel, the top and bottom are easily taken to pieces for cleaning purposes. They are the best I have seen for the pocket, but are heavy, cumbersome, and expensive.

Various kinds of bedside spittoons have been invented, but nothing is better than the old-fashioned common hospital pattern of a crockery-ware mug with a movable collar; they have the advantage of being easily cleansed and of allowing the medical man to inspect their contents without difficulty—a matter of much importance, especially in cases liable to hæmorrhage. In private work spittoons with paper linings (known as cuspidores), are advisable; the latter cost about 11d. per dozen, and permit of the easy burning of the sputa in an ordinary fireplace without unpleasantness.

Aluminium spittoons of various patterns are made; they have the advantage of lightness in weight, averaging only $2\frac{1}{2}$ oz., but the nurse must remember that aluminium will not boil in hard water, nor can it be washed in soda and water without oxydising and so spoiling its appearance.

Some fluid disinfectant should always be kept in the spittoon (whether the open or flask variety be used) in order to keep the expectoration damp, and thus prevent the danger of the patient sowing broadcast dust laden with germs of tubercular disease. As a rule the medical man in charge of the case will give his own directions as to which disinfectant he prefers, but in the absence of such orders the nurse may use Lawes' Fluid 1 per cent., which has little or no disagreeable smell, or some other pine emulsion. Some authorities advise carbolic solution 5 per cent. strength, or perchloride of mercury, 1 in 1,000. The nurse should always place whatever fluid is chosen into the spittoon or flask herself, both with a view to its being really there in the right strength and quantity, and also to enable her to calculate exactly the amount of expectoration from each individal patient in the twenty-four hours.

In writing for nurses it should hardly be necessary to draw their attention to the careful and conscientious cleansing required for these flasks and spittoons. In a sanatorium the pocket flasks must be collected as a routine duty every night, when the patients are provided with spitting mugs for the bedside, cleaned thoroughly in a manner to be presently described, taken to pieces and left soaking in the disinfectant all night. They should also be boiled for ten minutes once a week. Dettweiler's flasks are most easily cleaned by unscrewing the top and removing the collar-piece with funnel attached, and the lower lid. Water from a tap should then be run through the glass body over a sink reserved for this purpose alone. When apparently clean the inside of the flask should be well brushed with an ordinary bottle brush, using the approved disinfectant. The nurse must always carefully note the quantity and character of the sputa, reporting at once to the medical man the slightest trace of colour observable in it, and reserving the same for his inspection. As a general rule she should keep the patient quietly in bed, under such circumstances, in a recumbent position, and forbid all attempts at conversation until his arrival. It will usually be found that patients themselves are very nervous as to signs of hæmorrhage and at once draw their nurse's attention to anything of a doubtful character.

In this institution every patient on arrival is provided not only with flask and paper handkerchief but also with a pocket of stout calico for their reception, which he is taught by the nurse to fasten inside his ordinary pocket by means of safety pins. The pockets made for the men are oblong in shape, 8 in. by $6\frac{1}{2}$ in., with an opening of 6 in. in the longer side. They are designed for use inside either the breast or side pocket of a jacket.

The women's pockets follow the pattern of that in a lady's dress or skirt, inside which they are intended to be placed. The measurements in this case are—width 6 in., height of deep side 8 in., short side 5 in., giving a slope of 6 in. to admit the hand.

The patients have strict injunctions to reserve these pockets for the appointed use only; they are boiled every week, and are designed to do away with the risk of infection to the patients themselves, as well as to others, from dried particles of sputa adhering to the dress or coat pocket.

With the same object in view our resident medical officer has quite recently caused small basins to be procured for the reception of the substitutes for handkerchiefs at night. We find the small 4 in. "White Navy" bowls answer the purpose admirably; they have the great merit of compactness, and being enamelled are unbreakable and easily cleaned. They are also inexpensive, some houses quoting them as low as 2s. 8d. per doz. These bowls, together with the spittoon, stand on the locker by the side of each bed, and have completely done away with the old and dangerous habit of placing the handkerchief under the pillow, from the warmth of which the expectoration was almost certain to become dried and "dusty," and a source of infection to all in the room.

### Appointments.

SALOP INFIRMARY.—Miss Annie Bramwell has been appointed Night Sister at the above infirmary. She was trained at Charing Cross Hospital, and the City of London Lying-in Hospital, and has the L.O.S. diploma. Miss Bramwell has since held the post of charge nurse at the Park Fever Hospital, Hither Green.

### The History and Development of Trained Nursing.

### I.—THE DAWN OF NURSING.

If trained nursing, regarded purely from the professional standpoint, has reached a fuller development in the hands of its able organisers in the United States than in this country, yet it is generally recognised by those who have most closely studied the evolution of the nurse, and watched her at her work in both hemispheres and in many lands, that the very best of training is still to be found in English hospitals.

The present seems a not inopportune moment to remind English nurses of the fact that, though to Pastor Fliedner and his Training School for Deaconesses at Kaiserswerth must most ungrudgingly be given the credit of the first beginnings of the great movement which has resulted in the establishment of a new and noble profession, yet the rapid spread of trained nursing all over the world is undoubtedly due to the example and initiation of their countrywomen, and first and foremost to Florence Nightingale, its great pioneer. At the forthcoming International Congress of Women the official representation of nursing amongst professions is being left in the hands of ladies from the States, the Colonies, and foreign countries, and, having regard to the somewhat obvious inference of this arrangement, it may be interesting briefly to recount once more the history of modern sick nursing and to consider its progress.

It should never be forgotten, in that spirit of self-satisfaction which is so marked a trait of our progressive age, that although the nineteenth century has seen the birth of the systematically trained nurse, yet nursing of a high order had been known for hundreds of years past in the hospitals and institutions under the devoted care of religious communities. Tendence of the sick from the earliest days of Christianity was one of the chief duties undertaken by religious orders, deaconesses, monks, and nuns, and on the Continent throughout the Middle Ages noble work was done in hospital and on battlefields. In those far-off days it was a labour inspired solely by the love of God and of humanity, and performed as a religious act, often with supreme self-devotion. The paid lay nurse has been a product of comparatively recent times.

In the early years of the present century, whilst in the Roman Catholic countries on the Continent the nursing of the sick remained in the hands of Sisters of Mercy, and was quite innocent of any system worthy of the name, in England the condition of affairs was deplorable. The dissolution of the religious houses and scattering of the Roman Catholic orders had led to utter disorganisation in the administration of charitable institutions, which prior to that upheaval were in the hands of these orders. And when in the seventeenth and eighteenth centuries hospitals as we know them began to arise, nursing in any real sense of that word was simply non-existent. The women who did the work in the wards were of the lowest character as a rule, though no doubt there were bright exceptions. "Before 1840," Sir Henry Burdett says in "Hospitals and Asylums of the World," "nurses were almost the worst set of working women; the only points to be settled on engaging a nurse were that she was not Irish and not a confirmed drunkard." "We always engage them without any character," wrote a doctor, "as no respectable person would undertake so disagreeable an office." "Every vice was rampant among these women, and their aid to the dying was to remove pillows and bed-clothes, and so hasten the end." But at this very time the spirit of reform was stirring in England, as in Germany, and the year 1840 saw the foundation in Osnaburgh Square of the first nursing institution in London by Mrs. Fry and Lady Inglis, under the patronage of Queen Adelaide, and at the suggestion of Dr. Gooch and Robert Southey. It is possible that Elizabeth Fry was stimulated in this direction by the visit to London in 1832 of Theodore Fliedner, who was then maturing the scheme which resulted in the opening at Kaiserswerth in 1836 of the Deaconess Institution, where Florence Nightingale and so many pioneers of the nursing movement received their training. The sick poor had "lain long upon his heart," we are told; "there were many towns without a hospital, and there were hospitals without nursing"; he saw one in England "gleaming with marble," but there was "miserable tending of the body." Mrs. Fry visited Kaiserswerth herself later, and her name should ever be honoured among nurses as that of the first pioneer in nursing reform in England. Her institution for "Nursing Sisters" is still in existence in Devonshire Square, Bishopsgate.

But progress was slow, and in these days of eager volunteering for any and every kind of work, it seems almost incredible that when, in 1847, Sir Edward Parry issued a request for nurses for Haslar Naval Hospital, to be trained for six months at the German Hospital, Dalston, and at Kaiserswerth, not one response was received. The fulness of time was not even yet, though very close at hand. Sairey Gamp and Betsy Prig, whose immortal portraits were given to the world by Dickens in 1844, still reigned in the sick room; but the revolution against their terrible rule had begun in earnest.

In 1848 St. John's House was founded by Bishop Blomfield, of London, Dr. Todd, of King's College Hospital, and Mrs. William Morrice, as a home for the training of nurses for the poor, to consist of a society of ladies giving voluntary help and money, to be called "sisters," besides nurses of a lower rank receiving pay. Simultaneously, Miss Sellon began her work at Devonport, and a year or two later the Wantage Community was founded by Miss Lockhart, the new vigour stirring in the Church of England quickly leading to the establishment of the Clewer Sisterhood, and many other Anglican communities, all turning their attention to the improvement of nursing as a principal object. Training, as understood by the nurse of to-day, was of course not to be had. To Guy's Hospital Mrs. Fry's Nursing Sisters went for short periods; the first attempt at training on record. The St. John's House nurses were sent chiefly to Westminster and Middlesex Hospitals, going daily for a few hours, a short training to be followed by experience amongst the poor. King's College Hospital was also at this time endeavouring to aid the work of reform, and Miss Byron, who trained there in 1851, established a Home for Incurables in Margaret Street, from whence sprang the present Nursing Sisterhood of All Saints.

Once more we see the work of reformation began from religious motives, and in those times, when it was indeed an act of courage to enter a hospital, nursing was truly a "calling" which brought forth to their utmost the qualities without which a woman may be scientifically trained, but can never be a "nurse" in the sense understood by Florence Nightingale and her like.

Meantime, Florence Nightingale had been gradually preparing herself for the great task which lay before her. Twice she went into training at Kaiserswerth, spending some time also amongst the Sisters of Mercy at Paris, and devoting her unusual mental powers to a thorough study of the question of hospital nursing, construction, and administration. Of Kaiserswerth, Miss Nightingale says in an autograph letter which is preserved in the British Museum, "I was twice in training there myself. Of course, since then hospital and district nursing have made giant strides; indeed, district nursing has been invented, but never have I met with a higher tone, a purer devotion, than there. There was no neglect. It was the more remarkable because many of the deaconesses had been only peasants; none were gentlewomen when I was there. The food was poor. No luxury but cleanliness."

### Typical Patients.

### THE CHEERFUL PATIENT.

He came to the hospital for the purpose of undergoing a most critical operation. His club surgeon had told him that he was suffering from kidney trouble, and that there was nothing for it but the knife. He had had many seizures—one lasting thirty hours—and for nearly two years had scarcely known what it was to discharge his day's work or even walk about without more or less acute pain. Yet he was cheerful on arrival, and volunteered to walk up to the ward instead of being carried up by a couple of porters in an invalid chair. He told the sister that he had been with a City warehouse firm for over twenty years, that he worked from eight to seven every day except Saturday, that he had a wife and ten children, that he was fifty years old, and had been fortunate enough not to have had a single day's illness until the present trouble, and that he looked forward to a successful operation and a return of robust health and daily toil.

"You seem to be an active sort of a chap; I guess you were born for work," said the low-spirited man who occupied the bed on his left.

"I believe I was, and I'm glad of it," was the cheerful one's reply.

"That's why you're put on a light diet; they like to see you picking the bones out of the fish. Now I'm put on fluid diet; my meals ain't any recreation to me, they're so soon over."

"Well, I'll read to you."

And the cheerful one would read by the hour to the patient on his left. The latter accepted the favour none too kindly; yet what a sight to see. A couple of patients in a hospital ward, one just about to be placed on the operating table and his life held to death's brink, and the other suffering from a merely troublesome ailment, yet the former striving to chase away the shadows, laying himself out to please, seeking to inspire hope and confidence.

"This time six weeks I shall perhaps be well and strong again. I was only talking to a friend the other day who told me that he knew someone who once had just the same complaint as I've got, and who was operated on and who quite recovered."

"But what if you don't get over it?" answers the gloomy one.

"Why, I suppose that, like a great many others, I shall join the majority before I reckoned on it."

"Then what'll the missus do?"

"She'll do her best! There's the club money that will more than cover the expense she'd be put to, and we've a good many friends who would do what they could for the youngsters. My eldest boy is eighteen now, and he's in a rising berth; he'd never see his mother starve, I lay."

"You're a fine plucked 'un, but I can't help thinking that it's only want of real experience of the world that makes you so light-hearted and cocksure."

So the stray conversation between the cheerful one and his neighbour runs.

When the surgeon makes his rounds he always finds hope and faith and patience writ large on the brow of the cheerful one. If a brief exterior examination of the affected part results in a terrible twinge of pain it is heroically borne, and there is no doubt in his mind as to the examination being necessary. There is no grumbling about the monotonous diet, no vain pleading for out-of-the-way privileges, no shirking medicine—just a certain trust that everything is for the best and that in due course all will be right again. The day of the operation arrives. The anæsthetic is well taken, the cruel stone is removed, everything is successful, a good constitution and a fine determination come to the rescue, and the battle with disease is won.

### In Memoriam.—Louise Darche.

Miss Louise Darche was born August 20th, 1852, at the village of Lampton Mills, near Toronto, Canada. On the death of her father, when she was eighteen, she qualified herself for a teacher, and for some years she was principal of St. Catherine's High School, Toronto. But her health, never very strong, compelled her to resign this position, and being advised to adopt a calling that involved more physical exercise, she entered Bellevue Training School for Nurses, New York. On the completion of her training she had charge of the son of a Chicago millionaire, and after the lad's death, the father, in gratitude for her services and in appreciation of the value of training for the work of a nurse, founded a training home for nurses at Pittsfield, Mass. Another of her private patients was a little girl with double hip disease, to whom she acted both as nurse and governess for a considerable time, until the child was able to walk without crutches. In 1887 Miss Darche was asked to undertake the charge of the training school for nurses on Blackwell's Island, New York City, and consented on the condition that she had a free hand in the choice of her subordinates. This being agreed to, she obtained the assistance of Miss Kimber, who thenceforth became her inseparable fellow-worker. On January 1st, 1888, the two friends entered on their arduous undertaking. The institution was at that time in a state of the utmost disorder and inefficiency, and had so bad a name that it was difficult at first to get suitable candidates to come forward for admission. Miss Darche's perseverance and power of influence triumphed over all obstacles, and in a few years her nurses were in request for the accident hospitals of New York, as well as the Central Charity Hospital to which the nursing school was attached, so that by degrees she came to have a general superintendence over all the hospitals that came in the department of the Charity Commissioners. The work was, of course, very laborious and anxious, and she could never be induced to spare herself where the interest of the hospital, nurses, or patients was concerned. It is not wonderful that her health, never robust, broke down, and her nerves gave way under the constant strain, so that in ten years, at the end of 1897, she was obliged to resign her post. After six months' rest with her friend in the home of her sister in Canada, it was thought that the change of climate and scene involved in a voyage to Europe might be beneficial to her. The movement and excitement of London, however, seems to have been too much for the over-wrought nerves, and her end, though a terrible shock to her friends, was but the climax to the gradual progress of disease. Her funeral service was held on Tuesday at St. Saviour's Church, St. George's Square, S.W.

### Where to Go.

St. John's Hospital, Morden Hill, Lewisham.—June 14th, at five p.m., laying the foundation-stone of the Lewisham Hospital by H.R.H. the Duchess of Albany.

Brassey House, 24, Park Lane.—Monday, June 12th, three p.m., Central Bureau for the Employment of Women. Annual meeting, Earl of Dudley in the chair.

### To Nurses.

In order to increase and vary the interest in the *Mirror*, we invite contributions from any of our readers in the form of either an article, a paragraph, or information, and will pay a minimum of 5s. for each contribuion. All payments are made at the beginning of each quarter, *i.e.*, January 1st, April 1st, July 1st, and October 1st.

### National Health Society.

ON Saturday the Duke of WESTMINSTER presided at Grosvenor House over the annual distribution of diplomas, medals, and certificates to the National Health Society's students, the presentations being made by Her Royal Highness Princess Christian. After the presentation the Bishop of London gave a short address, and concluded by moving the following resolution: "That this meeting cordially approves the objects of the National Health Society and of the methods by which they are being carried out, and congratulates the society on the results obtained from its comprehensive system of instruction." Sir William Broadbent seconded, and speeches in support were made by Dr. Farquharson, M.P., and Colonel Sir Herbert Perrott, Bart. Other speakers were Sir Richard T. Thorne, K.C.B., Sir Ralph Thompson, K.C.B., the Rev. Prebendary Ridgeway, and Mr. Frederick Treves, F.R.C.S. Universal testimony was borne to the good and useful work done by the National Society and to the very considerable influence exerted through its means all over the country on behalf of sanitation and hygiene.

### Hospital Fete at Hampstead.

IN aid of the building fund of the Hampstead Hospital and Nursing Institute, whose existing premises are most inadequate, a garden fête was held last Thursday under very auspicious conditions at Golder's Hill. The blazing sun made the rural character of the scene especially delightful, whilst numerous indoor and outdoor entertainments attracted many interested spectators. H.R.H. Princess Christian of Schleswig-Holstein, attended by the Baroness von und zu Egloffstein, drove from Buckingham Palace, and after lunching with Mrs. Gordon at Oakhurst, West Heath, arrived at Golder's Hill shortly after three. She was looking well, but pale, and was dressed in dark blue crepon relieved by heavy white lace, and wore a white feather boa. She was received at the entrance by Mr. W. F. Malcolm, the hon. treasurer, and Dr. W. Heath Strange, the founder of the institution, whilst a number of ladies interested in the work were assembled to welcome her in the hall. On passing to the balcony a bouquet was presented to her by Miss Leslie Hannaford, and a specially-bound copy of Mr. J. E. Whiting's book on Golder's Hill by Miss Swinburne-Hanham, granddaughter of Sir Spencer Wells, to whom the property at one time belonged. Numerous purses and cheques were next handed to the Princess, amounting in value to about £900. The Royal party then witnessed the very pretty musical bicycle ride. The young lady riders, dressed in white, and carrying bouquets on their handle bars, kept time and order admirably. In commemoration of the Queen's eightieth birthday Princess Christian planted a tree in the grounds, thus helping to keep up an ancient and pretty custom. After this ceremony tea was served at the house, and the Princess left for town.

A succession of dramatic and musical entertainments were provided during the afternoon in the billiard-room and drawing-room under the management of Mr. Bernard MacDonald.

In the billiard-room Mr. MacDonald was assisted in rendering a couple of drawing-room pieces by Mr. Charles B. Burnaby, Miss Beatrice Webb, Miss Mabel Terry Lewis, and Mr. Hamilton Fyfe. Miss Julia Neilson sang a couple of songs, Mr. Fred Terry gave recitations, and Miss Marianne Cadwell a monologue.

In the drawing-room the chief attractions were provided by Mrs. Kendal, who looked remarkably well in a pale grey moire antique dress relieved with white, Miss Genevieve Ward, and Miss Beatrice Herford.

Out of doors the Bicycle Gymkhana excited the most attention, but there was also a Punch and Judy Show on the lawn, and the bands of Her Majesty's Scots Guards and of the pipers of the 1st Battalion Scots Guards discoursed excellent music.

We hope that a substantial sum towards the £30,000 required—of which £4,000 has been raised—was realised by the fête.

### The Matron's Corner.

### STUDY OF CHARACTER.

MAY I say, "to those about to become matrons," as well as to those who already occupy that post, "study human nature"? It is a rule that may be safely laid down for every woman who wishes to be a good nurse. For a matron it is a *sine qua non*, because a matron has to deal with human nature in such varieties of form with so many types of character.

If through all her years of training a nurse tries to exercise to the full her powers of imagination, which enable her to understand and get behind characters unlike her own, she will find, when she is in a position of authority, that the knowledge she has gained of her fellow-beings is an inestimable boon. More patience, more tact, more loving observation are needful to understand a character unlike your own than are necessary to the understanding of hospital organisation, nursing, or housekeeping. It is a study like all other studies, and a difficult one withal; but the attainment of a knowledge of character is worth an infinite amount of pains. How often a matron may be a splendid organiser and yet you find her repeatedly striving to fit square people into round holes, simply because she lacks true perception of temperament. Is it not often the case that such and such a probationer does excellently in this ward, very badly in that, yet for no obvious reason? May it not happen that it is nobody's fault, but that the temperaments of the sister and probationer clash hopelessly?

You may answer, "It is impossible to consider whether my probationers' temperaments are suited to this ward or that." But is it impossible? Without carrying the matter to any absurd lengths, is it not the truest wisdom to try, if possible, to fit your subordinates into the places best suited for them? (Why put a shrinking, nervous probationer into the ward of a sister who is by nature a bully, for example?)

There must sometimes be friction in a hospital; but surely the less there is the better for both patients and nurses. After all, a hospital is not a penitentiary. You do not wish to give your nurses the tasks most uncongenial to them. A wise ruler studies the characters of his subordinates, and allots to each the place for which he is most suited. Surely a wise matron also tries to draw out the abilities and powers of her staff—discovers their capabilities and places them accordingly.

This implies real work and study—it is a striving after the ideal way of ruling a hospital. It entails much patient observation, much sacrifice of your own leisure; for to know your staff thoroughly you must know them in their leisure, as well as in their work. And even if the ideal seem so high as to be impossible, it is the striving after an ideal which is the main thing, for as Browning has it—

"The end, if reached or not, makes great the life."

May not this be said of institutions as of persons?

### Wants and Workers.

MISS MAY BLENKHAM, Laurel Bank, Dacre Hill, Rock Ferry, is desirous of obtaining surgical aid appliances and invalid chairs for the use of the recently inaugurated Sunshine League. This league is intended to brighten the lives of the cripple children in Birkenhead and the neighbourhood, and help in the manner suggested will be much appreciated.

NURSE SEATON, Ashstead House, near Epsom, offers her services free to a fever hospital for three weeks in return for the experience to be gained.

### Echoes from the Outside World.

### AN OPEN LETTER TO A HOSPITAL NURSE.

SOME natures seem capable of retaining their faculty for enjoyment to the end. I was much struck, in reading the account of the doings at Balmoral, to note that the Queen not only took the opportunity of ordering Pinder's Royal Circus to give a performance to her tenantry, servants, and officials, but, in her little pony carriage, drove to the show herself and remained for about an hour, appearing much amused at all that went on. The incident is another indication of the simplicity of her taste, notwithstanding the ceremonials of royalty which naturally hedge her about, and it also displays her thoughtfulness for others. Her dependents unquestionably thought twice as much of an entertainment good enough for the Queen to patronise, and as for the performers, all their lives the honour will cling to them that they appeared before the Queen of England and Empress of India.

---

POOR Queen Regent of Spain! I am so sorry for her. It may be perfectly true that worse colonists than the Spaniards never existed; that their only idea of making a successful colony was to take all they could out of the land, and to care nothing for the welfare of the subject races; and that had not the Caroline Islands, Palaos, and the greater part of the Ladrones been voluntarily given up to the Germans for a substantial consideration, there would have been a serious rising in the Islands to contend with, without the necessary forces at hand to quell it; but the fact remains that it must have cut Queen Christina to the heart to speak as she did to her people on the occasion of the opening of Parliament. The words, strong in dignity and suffering, are intensely pathetic. "At the opening of these Chambers, all the sorrows which have afflicted our hearts in consequence of the misfortunes of the country are renewed. Nevertheless, it behoves us to retain our sadness that we may derive some profit from experience." Later on she confesses that she believes "silence will avail them more than lamentations," and ends by exhorting her people "during peace to display the same resignation which they showed during the war, for," she adds significantly, "the times are critical." The Queen's loneliness, anxiety, and grief must be very hard to bear, and her life must be one continuous prayer that she may be allowed to hand over to her son, should he attain his majority, at least the inheritance his father left, Beloved Spain, even if it should be shorn of its foreign possessions!

---

IT is difficult for us, who are living in the midst of plenty, to realise that it is possible for 5,000,000 people at the present time to be dying for the want of a loaf of bread. And yet in the Eastern provinces of Russia matters are as bad as they well can be. Food has been scarce for some time, now it is failing entirely, and worse than all, scurvy, typhus, typhoid, and other diseases in their most aggravated forms are spreading quickly amongst the starving people. Scurvy is so little known amongst outsiders like myself—though nurses have of course to nurse it occasionally—that it needs a description like the following to enable us to grasp its horrors. A pastor who has seen the sights with his own eyes writes:—"In many houses five, six, seven, or even eight people are lying in rows on the broad benches which serve the Tartars as beds, all practically naked. On our entrance they simply bared their teeth, which were loose and bleeding, to show that they could not speak, and pointed to their legs, which were crippled with pains and covered with sores, to show that they could not rise to greet us." It appears that scurvy, as well as causing the teeth to loosen and decay, the gums to inflame and the tissues to decay, makes the blood vessels of the mouth swell and rupture. Fancy under these circumstances having nothing to live on but a few pieces of hard black bread, which "burst the inflamed arteries of the mouth, shake out the loosened teeth, and give excruciating pain." And yet even this most unsuitable nourishment will soon not be forthcoming unless those whose hearts are tender, even if their purses are light, do not come to the rescue. The Emperor and Empress, the Government, the Red Cross and other philanthropic societies, and many individuals have subscribed millions of roubles; but it is yet three months to harvest, and unless the English will help, as the Russians helped us at the time of the Indian famine, thousands of men, women, and children must perish miserably. Devoted Russian ladies at their own expense, mothers, medical students, even musicians, have gone to the rescue, many of them spending their holiday in thus ministering to the sick, but none of them are women of means, and without money they can do nothing. Three shillings, with rigid economy, will feed a man for a month. Even this may be too much for some of us to spare, but surely most of us would deny ourselves to the extent of 1s. 6d., and so save a human life for a fortnight. If all who read this would forward a donation, however trifling, to Mr. J. T. Woolrych Perowne, 3, Bryanston Place, W., the honorary treasurer in England, it would be no insignificant sum which could be sent out to St. Petersburg, and I am quite certain we should feel the happier as we sit down to our own board, spread amply with food, to feel that we had done what we could to give the cup of cold water, to which so rich a blessing has been promised. I may add that Sir Capel Wolseley, Bart., a member of the English Famine Committee, is leaving St. Petersburg to superintend the administration of the relief fund in the centre of the famine district. He will, I understand, have charge of the central store of provisions and medicines, and report periodically to the Rev. A. Francis, his chairman. He is accompanied by a volunteer party of workers, two of whom are English ladies.

---

ALL who love dancing and all who love music must alike grieve that Johann Strauss—whom the Viennese called "The Waltz King"—will never compose again. Millions of feet have rhythmically moved with more or less delight to the strains of his wonderful waltzes, and those whose dancing days are now well nigh past will remember the extraordinary popularity which was once achieved by "The Blue Danube"; how every orchestra included it in their repertoire; how every dance list showed it in large letters; how every pianist played it; and every good dancer asked for it wherever they had the opportunity. And yet, strange to say, Herr Strauss never learnt dancing, never waltzed in his life, and composed on a harmonium, the least suitable instrument for festive strains. His life was a practical demonstration of the saying that "Fear seldom kills." He was a victim to nervous disease, always afraid of catching an illness or of suffering from the complaints which he heard his friends had contracted. Yet he lived to be seventy-four years old, and died from what was a "simple cold" in the beginning, caught, it is said, by his own carelessness.

---

I HEAR that a member of the House of Commons appeared in a new style of chimney-pot hat the other day. Instead of its being made of black silk, it was made of straw! The effect must have been strange, but the extra comfort must have been great, and the innovation will probably spread. If you use your eyes you will possibly, ere long, see one in the London streets.

### Everybody's Opinion.

[Correspondence on all subjects is invited, but we cannot in any way be responsible for the opinions expressed by our correspondents. No communication can be entertained if the name and address of the correspondent is not given, as a guarantee of good faith but not necessarily for publication, or unless one side of the paper only is written on.]

### THE NURSING OF KAFFIRS.

"A South African Matron," M.R.B.N.A., writes: I cannot let the remarks made by your South African correspondent in The Hospital "Nursing Mirror" of April 15th on the nursing of Kaffirs by "cultivated women" pass without a strong protest. Looking back through nine happy years of hospital life in South Africa as pupil, staff nurse, ward sister, and matron, the time spent in the native wards stands out as the pleasantest, most interesting, and most instructive of my experiences. Well do I remember my first introduction as pupil to the native surgical wards in Kimberley Hospital, and the three boys with injured eyes who were my special care; how grateful they were when I established them in some warm sheltered corner. It was winter time. They always knew my step, and would follow me in and out of the ward catching hold of one another's "Josses." I recollect how interested I was in them, and how I rejoiced in the partial recovery of the sight of two of them; and later on when I was alone on night duty and a native was dying, I remember a Christian native patient keeping awake all night because he did not think it right I should be left alone with a dying boy. Still later, I recall how, when I was breaking down after some years of hard work, and was in charge of a large convalescent native ward, containing from sixty to seventy patients, I had told them they must be good and do their work without giving me any trouble as my head was bad, and how quietly they washed up their plates and beakers, swept out their wards, &c., without needing another word from me. Looking back, case after case comes to my mind of gratitude, kindly thought, refinement, and humour shown by my native patients, both male and female. I am sure some of your readers who were old Kimberley nurses will remember "Ole Bovi," who was the embodiment of all these traits, and a thoroughly sincere Christian as well. Only yesterday I was presented with sixpence from a very scanty store by a native patient who was leaving us; it was almost the only thing he had to give away, and meant a lot of gratitude. That the natives have their faults I do not deny for one moment, but they are the faults of children, and everyone who has had much to do with them in hospital wards and has treated them with consideration will bear me out when I say that as a rule they make most amenable patients, and are capable of being trained into good and faithful servants.

### METROPOLITAN ASYLUMS BOARD HOSPITALS.

"A. E. D." writes: I cannot tell you what pleasure it gave me to read "K. M.'s" letter to-day. I was at the North-Eastern Hospital for twelve months and can truthfully say they were amongst the happiest I have ever spent. I consider that fever training is just as essential to a nurse as general training. I learned many things while I was there, the principal ones being method and thoroughness. The matron and doctors were everything that could be desired, and the lectures excellent. No pains were spared to provide amusement for the nurses and their comfort was always studied. I could not resist to say a word in favour of a hospital where I had been so happy.

"A Word of Warning" writes: Will you permit me, another old nurse of the Metropolitan Asylums Board, to join in the praise given to the fever hospital. I, too, as charge nurse, spent a very happy time there, and shall always feel grateful for the very kindly consideration shown me by the matron and medical staff. But I should also like to call attention to the fact that all fever hospitals are not like those named by your correspondents. I left the one in which I worked and went to another under the same board of management, but what a difference! Absolutely no consideration shown or interest taken in the nurses, either on or off duty. It was a splendid place, the accommodation good but the nurses (with few exceptions) stayed only a few months and left thoroughly disgusted with fever hospitals. I agree with "K. M." that no nurse should consider her training complete without fever experience, but let her make careful inquiries before entering any institution. I feel I must send a word of warning to your readers, lest some may be misled by the glowing accounts that have appeared in The Hospital of the last few weeks.

### PRIVATE NURSES.

"White Heather" writes: May I be allowed through the columns of your paper to offer a few reasons for the discontent of private nurses? I think, in the first place, it is because they are so independent and expect too much from those who have engaged their services; and in the second because they look upon nursing far too much from a business point of view. A woman who through adverse circumstances is obliged to earn her own living should choose a profession she really likes, with the object not only of earning money, but also of doing some good in the world. Now, nursing, of all professions, should be regarded in this light, for how can a nurse help to cheer up, amuse, or in any way do good to an invalid if money and her own liberty are her main ideas? A much higher ideal should be aimed at, and if every nurse would tend her patient in a kind, gentle, and sympathetic spirit, just because she loved the work she had taken up, and not merely as a means of livelihood, she would not in the future have to bemoan her life as a private nurse, but would be able to say truthfully she was blessed in her work and perfectly happy.

"A Nurse from the Emerald Isle" writes: I am very glad to see that the question of private nursing institutes has been so much taken up lately, and highly commend "Blue Bell" and "Lily" for their plucky remarks. I joined a nursing institute about six months ago, and during that time earned for the institute close on £60, and was paid in return at the rate of £32 a year. Not a shilling percentage was given me. The staff was small, and the treatment we received while in the institute was anything but home-like. We slept on hard, lumpy flock beds, with insufficient clothing, and that of the coarsest quality; did not even have a rug or carpet to step on upon getting out of bed, and were not allowed a drop of warm water in the depth of winter for our morning ablutions. Our rules were hard and fast. No such thing as breakfast in bed for the poor tired-out nurse coming in from a bad case. And in this same institute, if a nurse was ill or could not come down to breakfast, she was censured and made feel her po tion keenly by the superintendent or her deputy, who were hard-hearted women in every respect, and had no thought for their nurses beyond getting them out to cases directly they came n from one. I know one instance in which a nurse went to a case of influenza. While there she contracted the disease, and the medical attendant declared that with such a high temperature she was not fit to go on with her case. The nurse telephoned to the institute saying she was ill and could not go on with her work. The superintendent doubted her word, and telephoned to the doctor for an explanation. He at once answered back and confirmed the nurse's statement. Now this poor thing came back to the institute looking feverish and ill. She was questioned and catechised by the superintendent and treated as if she were "shamming." She was neither given a hot drink nor ordered to bed; she simply sat down until she could not keep up any longer, and begged leave to go to bed. That girl had to sleep in a bed which had not been slept in for some weeks, without even a fire to cheer the comfortless room, and had she not been looked after by her fellow-nurses I do not know what she could have done. When she became convalescent she was sent off to nurse scarlet fever. This institute was a new one, and was altogether conducted as a business, and strictly on a money-making principle. Nurses were chosen at random, and consequently the doctor's work and the patients suffered. Those nursing institutions which are conducted on the cooperative principle have a decided advantage over the others. The nurse gets the benefit of her work financially, and is certainly more interested in her cases, while her patients are the gainers, whereas a nurse working for a fixed salary has nothing but the love of the work to spur her on.

### "Ward No. 17."

It was a small ward with only two beds, but somehow it interested me more than all the rest of the corridor. The bed nearest the window was occupied by "Roberts," a tall, fine young fellow of about twenty-two, whose overflowing spirits and gaiety of manner made him a general favourite. He was suffering from an ulcerated leg, but expected soon to be discharged from the hospital. In the course of my duties in the wards I naturally became acquainted with the peculiarities of a good many of the patients, and soon found out that Roberts held high-flown notions about many things; the Bible he regarded with a kind of lofty scorn, as doing very well for feeble old women and children, but as quite an unnecessary factor in the ruling of his own life and conduct. Roberts was great at argument, and could talk glibly about the burning questions of the day, and, as his companion in the ward was a quiet, reticent man, he usually settled all matters under discussion entirely to his own satisfaction.

Old Santy in the other bed was the owner of an old-standing fractured leg, one of those obstinate compound fractures which are the despair of doctors and nurses. Santy refused to consider the advisability of a wooden limb, and stoutly declined to part with his leg even if he had to wear bandages for the rest of his life. Of course Roberts chaffed old Santy incessantly, and gravely informed him that he would be eventually purchased by a well-known London company and converted into "Potted Meat."

Roberts professed a lofty indifference to Royalty in general, and the Prince of Wales in particular, and when it became known that the Prince and Princess of Wales were likely to pass the hospital exclaimed, "Who's the Prince of Wales, I should like to know, with a 'ead like a skating rink?" but, in spite of his unflattering allusion to the growing baldness of His Royal Highness, instinctive loyalty rose to the surface, and he begged permission to get up and see the royal carriage pass, when doubtless he cheered the occupants as lustily as any of the other patients. But, alas! on that particular morning the ground was damp; Roberts took a chill, which rapidly developed into a high fever, with extensive inflammation of the tissues surrounding his wound.

The poor fellow's meekness and docility were now quite pathetic. "Oh, nurse, do read to me!" and the once despised Bible was brought to his bedside, and the quiet voice of the Nurse read the old and yet ever new story of the Prodigal returning from "a far country" to the Home of his Father. Roberts listened with the deepest attention, and the parable seemed to take a fuller meaning as it fell on the ears of the repentant prodigal who had been "into the far country," and now longed to go back to his Father's house. A few days more and Roberts was wildly delirious, and, without afterwards regaining consciousness, he passed into the "Silent Land."

The very morning after his death his only sister came to see him, all unconscious of the sad scene awaiting her. Words cannot depict her grief and despair which were painfully accentuated by the gay finery she wore; the poor soul wrung her hands and wailed aloud in the bitterness of her sorrow; no last words to soften the agony of parting, nothing but the memory of his bonnie face henceforth "through life's long day." Many sorrowful scenes have been woven into the thread of my hospital career, but nothing ever touched my deepest sympathies as profoundly as the sad ending of that bright young life, and the look of hopeless misery on the face of poor Roberts' sister.

### For Reading to the Sick.

### JESUS OUR LIGHT.

O blessed Lord! how much I need
Thy Light to guide me on my way!
So many hands, that without need,
Still touch Thy wounds and make them bleed,
So many feet that day by day
Still wander from thy fold astray!
Feeble at best is my endeavour!
I see but cannot reach the Light;
And yet for ever and for ever,
When seeming just within my grasp,
I feel my feeble hands unclasp,
And sink discouraged into night;
For Thine own purpose Thou has sent
The strife and the discouragement. —*Longfellow.*

The prescience of such souls has ever hailed,
Long ere the dawn, the coming of the Sun,
And may be, by such Faith the Light itself is won.
—*Houghton.*

He, who from the Father forth was sent,
Came the true Light, light to our hearts to bring,
The word of God; the making visible;
The far-transcending glory brought
In human form with man to dwell;
The dazzling gone—the power not less
To show, irradiate, and bless;
The gathering of the primal rays divine,
Informing Chaos to a pure sunshine. —*Macdonald.*

On Earth, Thou hidest, not to scare
Thy children with Thy Light;
Thou showest us Thy face in Heaven,
When we can bear the sight. —*Faber.*

Before the eyes of men let duly shine thy Light,
But ever let thy life's best part be out of sight.
—*Trench.*

The Light everlasting
Unto the blind is not, but is born of the eye that has vision. —*Longfellow.*

**Reading.**

"Faith cometh by hearing, and hearing by the word of God;" accordingly we read in verse 30 (John viii.), "As he spake these words many believed on him." So He taught and so they believed; as the Apostle puts it, "So we preached and so ye believed." It is always in connection with the word of truth that the Holy Spirit works in us. Christ's voice and the Spirit's hand go together. We find this in our text, but we find more than this.

The reception of Christ's word begins discipleship. There may be many an anxious thought before this; many a tear, many a bitter groan. There may be alarm, and disquietude, and inquiry. But these are not discipleship. They are but so many gropings after teaching. . . . All the world is in its poor, dark way, stretching out its hands after something which can only be realised in Christ. But this is not discipleship. All men are saying, Who will show us any good; but this is not discipleship. That begins with receiving His word; not with doing some great thing, but receiving His word; receiving it as the scholar receives his master's teaching. He is the word, and he speaks the word. . . . It is a word (1) concerning the Father, (2) concerning Himself. He comes as the revealer of the Father and as the declarer of Himself and His work. From the moment that we receive what He tells us concerning the Father and Himself we become His disciples, His scholars. Thus we are taught, not of man, but of God. This is the true, the authentic beginning of discipleship.—*Bonar*

### Travel Notes.

### XXVI.—A HOLIDAY IN SICILY.

By a Nurse.

Last autumn I spent a very pleasant fortnight at the ancient hill town of Taormina, on the coast of Sicily, midway between Catania and Messina. Starting from Malta in one of the Austrian-Lloyd steamers at 1.30 p.m., Syracuse was reached after a rough, but to me delightful voyage, at 10.30. Lights twinkling from the town and all along the marina was all that could be seen of the place as we slowly steamed into the harbour.

Very soon a crowd of small craft, manned by the general style of Mediterranean boatmen—noisy and quarrelsome fellows—came alongside and took passengers and their luggage ashore to the Custom House. After the usual examination I entered the carriage sent from the Villa Politi, the pension where I was to spend the night and join the friend who was going on to Taormina with me. A rather long and jolting drive along a dark country road at length brought me to the villa, the mistress of which—a hospitable German lady—gave me a warm welcome and an excellent cold supper, and after a chat with my friend I was glad to find the comfortable little bedroom reserved for me.

The following day we breakfasted in good time, and, after a ramble in the charming garden of the villa running along the edge of some old Greek stone quarries, we bade our hostess "adio" and drove off to the station. Our road skirted the town, so we only had a glimpse of Syracuse, which I am told is not an attractive place, though picturesque enough as seen in a bird's-eye view from the Villa Politi. It is decidedly an interesting town on account of its many ancient historical remains, Dionisius' Ear—which I regret we had not time to visit—being one of them. We had barely time to get our ticket for Giardini (the station for Taormina) and see our luggage weighed and booked before the train started for Catania.

A knowledge of Italian is, of course, extremely handy in travelling. French also is useful, as it is generally understood in the hotels and larger shops. Luggage should be reduced to the smallest possible amount as it is all charged for, except the light articles which can be taken in the carriage, and even these are seized upon by porters who demand preposterous tips for doing nothing, which one can do equally well oneself. The best way is to carry one's own bags and wraps and sternly refuse their attentions. After luggage is once booked it is seen no more till the journey's end. The railway carriages are comfortable and roomy, and fares are reasonable. The journey from Syracuse to Giardini first-class, taking five hours (not counting the hour spent at Catarina) costs sixteen francs, with luggage, a franc being equivalent to 9d.

One often associates Sicily with brigands, but when I broached the subject I was assured with a smile that nothing was to be feared from them nowadays, especially along the beaten track we were going. The country is pretty and fertile between Syracuse and Catania, and the first sight of Etna is obtained when half way, getting more and more distinct as the latter place grows nearer.

The part of Catania seen from the station struck me as remarkably ugly. The houses and roads being constructed of lava have a dingy and gloomy appearance, and the constant smell of sulphur is decidedly unpleasant. Comfort and cleanliness evidently are not considered necessary adjuncts to a Sicilian railway station, and there is absolutely not a single seat on the long dirty platform at Catania. When the Tressina train steamed in it was a welcome sight. We had a most enjoyable ride through delightful scenery of a very hilly character, covered with vineyards and orange groves, with Etna on the one hand, and the sea, hundreds of feet below, on the other. Giardini—an uninteresting fishing village—was reached at four p.m. Leaving the train we stowed ourselves and our luggage into a roomy but shaky old carriage, drawn by two equally shaky horses, and set off for Taormina, which is perched on the hills over a thousand feet above the sea, and reached by a capital road winding backwards and forwards for a distance of three miles. We entered the town, which was once strongly fortified, by one of the two gates, formerly the only entrances. The gates and the old walls, and in fact most of the other buildings, are in a very dilapidated condition, though it is evident even now that this city of the mountains long ago held a unique position of strength and dignity. On all sides departed greatness is illustrated by noble specimens of classic art, or fine palazzi and churches in the Sicilian Gothic style.

Everything (more or less) pays duty in Sicily, and our carriage was stopped and luggage examined by the Customs official. A drive of a few minutes through the principal and, practically, the only street, brought us to our hotel, which, though not the smartest to look at, was, as we afterwards discovered, as comfortable, quiet, clean, and airy as any in Taormina. The rooms were all that one could wish, the cooking excellent, and the scene from the windows, fittingly described by the name of the house, viz., Grand Hotel, Belle Vue. We had arranged beforehand about terms, and paid 7 frs. a day, including daily warm baths and fish or eggs for breakfast, these two items being charged for as extras unless specified to the contrary. Sicilian wine is put on the table at lunch and dinner free. Our window looked out on to a wide view of sea and hills, Etna lying to the right, with a cloud of smoke on the summit and a little snow in sunless ridges. The mountain at dawn and sunset often presents some marvellous effects of colouring.

We found the air delightfully bracing, and after a few days a decided increase in vigour and appetite was noticed. We spent our days in long rambles among the hills, along paths where mules and ponies were the only transport, but good pedestrians will find walking very pleasant and not fatiguing, the air is so fine. Sometimes we reached a ruined chapel or castle, and once went as far as the village of Inola, perched on its rock like an eagle's nest, and only reached by a precipitous mule track. A very intelligent boy, speaking French, guided us to what remained of the ruined Castello and the three churches of the village, which, despite its romantic surroundings, is about the dirtiest place one can imagine. Children, pigs, goats, and fowls all tumble about together in happy confusion. The hillsides round Taormina are covered with vines, almond trees, and the graceful olive, while wild flowers, blackberries, and ferns grow in profusion. Spring is really the best time for a visit, as the flowers are then at their best, but that being "the season," the hotels are full, and prices higher.

The great attraction of Taormina is the Greek Theatre, dating from 300 B.C., and even now a most interesting, unique, and extensive classic monument. It is one of the finest ruins of the kind extant, and great care is now taken to preserve it from further decay, though about 200 years ago some of its finest marbles were borne away to adorn the palace of a neighbouring duke, while up to a recent date, weeds and creepers were allowed to flourish and run riot over its columns, altars, and stairways.

The various charms of this "Sicilian paradise," as it has been called, caused me deep regret when obliged to leave it, and at the same time made me resolve to visit it again should opportunity offer.

I fancy the best way to reach Taormina from England is via Reggio and Messina. I may here mention that the whole of my expenses came within £10, and at the same time express a hope that these few notes may prove useful to anyone wishing to follow my footsteps.

NOTE BY THE TRAVEL EDITOR.

It must be borne in mind that the £10 which covered our correspondent's expenses would not be equally elastic if one visited Taormina from England. This lady only travelled from Malta. The great drawback to travelling in Sicily is the heavy initial expense of the journey. From England to Taormina, 1st class, is £14 16s. 10d. ; 2nd class, £11 2s. 6d. ; this takes the gilt off the gingerbread, but if you do not mind the expense, nothing is more charming than a tour in Sicily. Palermo is an ideal spot from every point of view.

### TRAVEL NOTES AND QUERIES.

RULES IN REGARD TO CORRESPONDENCE FOR THIS SECTION.—All questioners must use a pseudonym for publication, but the communication must also bear the writer's own name and address as well, which will be regarded as confidential. All such communications to be addressed "Travel Editor, 'Nursing Mirror,' 28, Southampton Street, Strand." No charge will be made for inserting and answering questions in the inquiry column, and all will be answered in rotation as space permits. If an answer by letter is required, a stamped and addressed envelope must be enclosed, together with 2s. 6d., which fee will be devoted to the objects of the "Hospital Convalescent Fund." Any inquiries reaching the office after Monday cannot be answered in "The Mirror" of the current week.

BRITTANY (Cyclist).—You can take your machine into France free of charge if you are a member of the C.T.C., but the authorities insist upon a plate being visible on the machine with your name and address. You must also obtain at the Customs a "Permis de circulation," cost 60 centimes. That is all you require.

NAPLES (Daffodil).—It would be much too hot at that season for most people's taste, though it is not so oppressive as in Rome. It would be better to go up to the mountains for the hot weather. A very long residence in Naples proves enervating to many. (2) Both Rome and Naples have a good water supply now. (3) It is during the hour before and after sunset that you should stay indoors. After that you may go out again without fear.

PAU AND ST. JEAN-DE-LUZ (Kettledrum).—The difference is chiefly in the sedative character of the climate at Pau. It is inland, and not bracing at all like St. Jean-de-Luz, but those who suffer from nervous headache would prefer it. Pau is a ville de luxe entirely, but St. Jean-de-Luz is much more primitive, and you are in lovely country in five minutes.

DINAN, BRITTANY (Economy).—It would be economical if you are content to live as the French live ; that is to say, without society and wholly without pretension. In that respect they are far wiser than we are—one servant where we should have two, and no entertaining. Living is cheap if you thoroughly understand the language, not otherwise. I think you might get furnished apartments, say, three rooms, for 20 francs a week, but you would have to hunt for them, and the sanitary arrangements are primitive. I had very good rooms there once. Three large rooms in the Rue de l'Apport for 35 francs a week, I remember, but they were really palatial. The English colony has somewhat diminished of late years, probably on account of its now being a large garrison town. For myself, I prefer it without the English.

CORNWALL VERSUS BRITTANY (Rifleman).—Yes there is a great similarity between the two climates and also in the scenery. For your purpose I should certainly prefer Brittany, the change would be so much greater. South Brittany is, of course, the warmer. As you are something of an artist, how would you like Pont Aven or Douarnenez?

SAN JEAN DE LUZ (Morning Star).—Yes, if you understand foreign living it can be managed very cheaply. You can live in apartments for £1 per week, but naturally to do this you must thoroughly understand the language and ways of the people. I speak from the personal experience of six months. Go to Hotel de la Poste—seven francs per day—and look out for rooms. I had four good rooms for 40 francs per month, *femme de menage* for 25 francs per month, and, of course, her food. There is a very nice English church and good English society.

A FORTNIGHT IN NORMANDY OR BRITTANY (Lena).—Take a return ticket to St. Malo, first, £2 12s. ; second class, £1 19s. 6d. Go to the Hotel du Centre ; ask for rooms on third floor. Spend some days there, and thoroughly explore the Bay of St. Malo as described in "The Mirror" two weeks ago. Then go on to Mont St. Michel, spending one or two nights there, Hotel Poulard Ainé. Next go to St. Briene, and sleep a night, Hotel de France, 7½ frs., more for only one night. This is not a particularly interesting place, but Brittany trains are very slow, and you could not get to Lanniore in one day. At Lanniore go to the Hotel de l'Univers. Stay there as long as you are interested, and make excursions to Perros, Guirec, &c. Then on to Morlaix, a typical Breton town, Hotel de Provence, 8 to 10 frs. From there you will see St. Pol de Leon, Roscoff, Ile de Batz, &c. Return viâ Quingamp, where break the journey, Hotel de France. An alternative trip would be to go down at once to Quimperlé and Quimper, but that involves much railway travelling, and your time is short. I think you might do the round I suggest in a fortnight very comfortably for from £10 to £12.

HEIDELBERG (North Inch).—The educational advantages are great, both for girls and boys, and living fairly cheap. The actual cost about the same as England, but the absence of all show, and the ability to see your friends without expensive entertainments, makes the difference.

SOUTH BRITTANY (Artist).—Your choice is excellent; supplement it with Douarnenez, so that you may study the Pointe de Raz and Baie des Trépassées. Your living should not exceed six francs per day until August. In August and September you must reckon for seven francs per day. Most decidedly Brittany rather than Normandy for your purpose.

*(For Travel Advertisements see Page* xvii.*)*

### Minor Appointments.

ROYAL HOSPITAL FOR SICK CHILDREN, EDINBURGH.—Miss Jean M. Wright was appointed Assistant Matron on May 29th. She was trained at the Western Infirmary, Glasgow. Subsequently she was for ten years sister at the Grimsby and District Hospital, first as night sister and then as sister of the surgical wards and theatre. Her next post was that of charge nurse at the Fountain Fever Hospital, Tooting; but for the last nine months she has been private nursing.

SMALLBURGH UNION.—Miss Ellen Maria Robbins has been appointed Nurse at Smallburgh Workhouse, and entered on her duties on May 15th. She was trained by the Meath Workhouse Attendants' Association at the Crumpsall Infirmary, Manchester.

### Notes and Queries.

**The contents of the Editor's Letter-box have now reached such unwieldy proportions that it has become necessary to establish a hard and fast rule regarding Answers to Correspondents. In future, all questions requiring replies will continue to be answered in this column without any fee. If an answer is required by letter, a fee of half-a-crown must be enclosed with the note containing the enquiry. We are always pleased to help our numerous correspondents to the fullest extent, and we can trust them to sympathise in the overwhelming amount of writing which makes the new rules a necessity.**

**Every communication must be accompanied by the writer's name and address, otherwise it will receive no attention.**

*Post Graduate Lectures.*

(98) I have had two years' hospital training, but am unable now to leave home. Could you kindly tell me through the "Nursing Mirror" of any lectures by correspondence, or other means by which one could continue to study? I take in THE HOSPITAL weekly, and also do some desultory reading, but having plenty of time wish for some more definite object, such as reading up some standard work, with the view to passing an examination on it. I should be greatly obliged if you or any readers of "The Mirror" could help me. Perhaps there are nurses situated like myself, who will appreciate the difficulty.—*Nurse.*

We should be glad if any of our readers can give "Nurse" information on this subject. There must be many nurses who would find much help and benefit from small, well-conducted reading societies.

*Rheumatism.*

(99) M. E. Glencoe writes in reference to Merci's query that if treatment abroad is not a *sine qua non*, the waters at Harrogate have generally good results in rheumatic cases. Also there are two nursing homes, both of which would probably be glad of nurses at the present time, so that the sister could be near her brother.

*A New Publication.*

(100) I am a maternity nurse, having taken my certificate at a London lying-in hospital. It has, however, fallen to my lot to help to nurse in medical cases, and I should be much obliged if you would tell me of a book on general nursing.

The "Manual of Nursing," by Laurence Humphry, would probably suit you.

*Return Expenses.*

(101) Would you be good enough to tell me what is the custom in such a case as the following: A nurse accepted an engagement to take charge of a lady in Rome, the agreement being that in addition to the fee her travelling expenses both ways should be paid. When the engagement came to an end the medical man in Rome asked the nurse to take another case for him. Does she forfeit the right to her return expenses? If she is now bound to pay them herself she will expend the greater part of her fee received from her second patient in getting back to England.—*X Y Z.*

The custom would have been to have claimed the expenses at the end of the case. It did not follow that you were to spend the money immediately on the purchase of the return ticket. Write and claim the amount due now, and if it be refused, and the game is worth the candle, engage a solicitor to arrange the matter for you. Don't, however, spend more in lawyers' fees than you are likely to get.

*Continental Institutions.*

(102) "Nurse" wishes for the names of Continental private nursing institutions or open-air sanatoria.

"Nurse" can hardly have read our query column, or she would have seen that we never recommend institutions.

*Private Nursing Home.*

(103) Will you kindly tell me of a small home of a good class where monthly and surgical cases are taken at seaside or in the country?—*Gill.*

We cannot take the responsibility of recommending institutions. We therefore refer you to the numerous advertisements appearing in the columns of the medical and other journals, advising you to be careful in the matter of references.

